# Knee Extensors Muscle Plasticity Over a 5-Years Rehabilitation Process After Open Knee Surgery

**DOI:** 10.3389/fphys.2018.01343

**Published:** 2018-09-25

**Authors:** Martin Flück, Claudio Viecelli, Andreas M. Bapst, Stephanie Kasper, Paola Valdivieso, Martino V. Franchi, Severin Ruoss, Jean-Marc Lüthi, Martin Bühler, Helgard Claassen, Hans Hoppeler, Christian Gerber

**Affiliations:** ^1^Department of Orthopedics, Balgrist University Hospital, University of Zurich, Zurich, Switzerland; ^2^Department of Orthopedic Surgery, Inselspital Bern, Bern, Switzerland; ^3^Department of Anatomy, University of Bern, Bern, Switzerland

**Keywords:** fiber type, mitochondria, focal adhesion kinase, loading, surgery

## Abstract

We investigated molecular and cellular parameters which set metabolic and mechanical functioning of knee extensor muscles in the operated and contralateral control leg of 9 patients with a chronically insufficient anterior cruciate ligament (ACL; 26.6 ± 8.3 years, 8 males, 1 female) after open reconstructive surgery (week 0), after ambulant physiotherapy under cast immobilization (week 9), succeeding rehabilitation training (up to week 26), and subsequent voluntary physical activity (week 260). Clinical indices of knee function in the operated leg were improved at 52 weeks and remained at a comparable level at week 260. CSA of the quadriceps (-18%), MCSA of muscle fibers (-24%), and capillary-to-fiber ratio (-24%) in *m. vastus lateralis* from the ACL insufficient leg were lower at week 0 than reference values in the contralateral leg at week 260. Slow type fiber percentage (-35%) and mitochondrial volume density (-39%) were reduced in *m. vastus lateralis* from the operated leg at weeks 9 and 26. Composition alterations in the operated leg exceeded those in the contralateral leg and, with the exception of the volume density of subsarcolemmal mitochondria, returned to the reference levels at week 260. Leg-specific deterioration of metabolic characteristics in the *vasti* from the operated leg was reflected by the down-regulation of mitochondrial respiration complex I-III markers (-41–57%) at week 9. After rehabilitation training at week 26, the specific Y397 phosphorylation of focal adhesion kinase (FAK), which is a proxy for mechano-regulation, was elevated by 71% in the operated leg but not in the contralateral leg, which had performed strengthening type exercise during ambulant physiotherapy. Total FAK protein and Y397 phosphorylation levels were lowered in both legs at week 26 resulting in positive correlations with mitochondrial volume densities and mitochondrial protein levels. The findings emphasize that a loss of mechanical and metabolic characteristics in knee extensor muscle remains detectable years after untreated ACL rupture, which may be aggravated in the post-operative phase by the deterioration of slow-oxidative characteristics after reconstruction due to insufficient load-bearing muscle activity. The reestablishment of muscle composition subsequent to years of voluntary physical activity reinforces that slow-to-fast fiber transformation is reversible in humans.

## Introduction

Skeletal muscle’s functional capacity demonstrates a pronounced dependence on the impact of use-related mechanical and metabolic stimuli (Booth and Thomason, 1991; Fluck and Hoppeler, 2003). This is amply illustrated by the marked deconditioning of the two main traits of muscle, strength and fatigue resistance, with reduced load-bearing muscle activity (Ferretti et al., 1997; Narici and de Boer, 2011; Li et al., 2013), and a partial reestablishment of the phenotypic hallmarks with the resumption of load-bearing activity (Desplanches et al., 1987; Pette, 2001; Zollner et al., 2012). The deconditioning of the muscle phenotype with prolonged disuse, during bedrest, plaster casting and unilateral suspension, involves as series of degenerative reactions in the muscle fibers and associated capillaries that change muscle’s metabolic and contractile characteristics [reviewed in (Muller, 1970; Fluck and Hoppeler, 2003; Adair and Montani, 2010)].

In the rat, the cellular reactions of anti-gravity muscle to disuse comprise the loss of slow oxidative characteristics and the reduction of mean cross sectional area of muscle fibers along with a shift from slow to fast type muscle fibers (Fluck and Hoppeler, 2003). These alterations may be accompanied by the rarefaction of capillaries (Adair and Montani, 2010), and collectively reduce aerobic capacity and the capacity for force production (Desplanches et al., 1987; Pette, 2001). Increasing contractile activity subsequent to disuse elevates the metabolic capacity of skeletal muscle by enhancing the volume content or density of mitochondria and capillaries, and capillary-to-fiber ratio, and reestablishes the normal distribution and cross sectional area of muscle fiber types [reviewed in (Desplanches et al., 1987; Pette et al., 2002; Fluck and Hoppeler, 2003)]. In a single example with young subjects it has been shown that slow-to-fast transformation also occurs in knee extensor muscle of human subjects with durations of immobilization as short as 3 weeks and can be reversed by subsequent strength training over a period of 12 weeks (Hortobagyi et al., 2000). The extent to which disuse-induced deconditioning of phenotypic muscle characteristics involves metabolic aspects, and the extent to which load-bearing types of muscle activity can reestablish the normal myocellular characteristics after longer durations of disuse is not understood (Howald, 1982; Fluck and Hoppeler, 2003; Wilson et al., 2012).

The molecular regulation of the two main traits of skeletal muscle by physiological cues has been shown to involve a network of signal transduction pathways, which govern, myogenesis and mitochondrial biogenesis (Hoppeler et al., 2011). A number of phosphotransfer enzymes (i.e., kinases) have been found as being instrumental in the upstream activation of signaling pathways upon the impact of load-bearing muscle activity (Hoppeler et al., 2011; Egan and Zierath, 2013). Amongst these the integrin-associated focal adhesion kinase (FAK) is an example for an upstream element which integrates fiber recruitment-related stimuli into the phosphorylation cascades that govern both traits at the level of gene expression (de Oliveira et al., 2009; Klossner et al., 2013; Graham et al., 2015). The phosphorylation level of tyrosine 397 of FAK serves as a proxy for its activation status [reviewed in (Klossner et al., 2013)] and is correlated to time under tension in rat muscle (Rahnert and Burkholder, 2013). Furthermore, expression levels of FAK are reduced with prolonged unloading while being increased with muscle overload (Narici et al., 2011; Li et al., 2013). In this respect, the expression and specific Y397 phosphorylation of FAK reflects the acute and chronic impact of mechanical loading and unloading (Fluck et al., 2002; Li et al., 2013; Rahnert and Burkholder, 2013; Graham et al., 2015). Thereby, increased amounts of Y397 phosphorylated FAK has been shown to promote, and correspond to load-induced transcript and, protein expression of mitochondria factors and slow type myosin heavy chain in rodents (Durieux et al., 2009; Franchi et al., 2018).

Alterations in skeletal muscle function are a yet poorly understood part of the musculoskeletal reactions to soft tissue injury. For instance, extensor muscle groups that operate on the knee joint present reductions in strength, after rupture of the ACL, leading to a hyperextension of the knee and instability of tibial rotation (Gerber et al., 1985; Palmieri-Smith et al., 2008; Hart et al., 2010). Surgical reconstruction of the insufficient ACL followed by physiotherapy and rehabilitation are practical options for the functional recovery of the operated knee joint (Saka, 2014). Physiotherapy aims to improve the range of motion of the joint and reduces soft tissue stiffness (Robertson et al., 2009; Panchal et al., 2017), while the objective of exercise rehabilitation is to regain muscle strength, improving fatigue resistance and stability of the reconstructed knee (Ardern et al., 2011; Notarnicola et al., 2016). However, despite best efforts during post-operative rehabilitation, deficits in strength and fatigue resistance often persist months to years after rehabilitative treatment ceases; thus remaining a concern when clearing the patients for return for professional or leisure activities (Konishi et al., 2002; Palmieri-Smith et al., 2008; Patras et al., 2009; Culvenor et al., 2013; Lindstrom et al., 2013; Otzel et al., 2015; Palmieri-Smith and Lepley, 2015). To date, it is not known to which extent the course of functional recovery of knee extensor function subsequent to ACL reconstruction is related to deficits in cellular hallmarks of muscle function and mechanically induced contraction-signaling.

The aim of the present investigation was to assess the degree of muscle transformation with chronic ACL insufficiency and the contribution and temporal sequence of adjustments in knee extensors muscle composition with rehabilitation after ACL reconstruction. As well we wanted to explore the relationships between such adaptations and alterations in clinical parameters of knee function and stability (Domnick et al., 2016). Specifically, we investigated to which extent cellular and molecular parameters of knee extensor function in operated leg, and its healthy contralateral control, would be affected by the surgical reconstruction of the ACL, successive rehabilitation, and self-motivated exercise up to 5 years after surgery (Konishi et al., 2002; Palmieri-Smith et al., 2008; Palmieri-Smith and Lepley, 2015; Notarnicola et al., 2016). The measurements included the morphometric assessment of muscle composition and the relative content of selected proteins involved in mitochondrial respiration (ATP5A1, COX4I1, COX4I2, NDUFA9, SDHA, and UQCRC1) and regulation of the biogenesis of myofibrils (myoD, myogenin) and angiogenesis (VEGF, tenascin-C). As well, the content and Y397 phosphorylation of FAK as a proxy for mechano-transduction was investigated. We hypothesized that the measure of selected key molecular regulators of muscle adaptation further resolves the different (active) phases of rehabilitation-induced musculo-skeletal plasticity. We expected that cellular adjustments defining the metabolic and mechanical phenotype would be inversely correlated to levels of expression and Y397 phosphorylation of FAK, and also be related to clinical indices of knee function and stability. The possibility of compensatory reactions subsequent to the surgical intervention (Lu et al., 1999) was controlled by comparing cellular and molecular effects in *m. vastus lateralis* from the operated leg to those from the contralateral non-operated leg. Special emphasis was put to compare the values to those in the contralateral leg 5 years of self-motivated exercise as this situation arguably is more closely related to normal values than those in legs with ACL instability (Gerber et al., 1985; Konishi et al., 2002; Palmieri-Smith et al., 2008; Palmieri-Smith and Lepley, 2015).

## Materials and Methods

### Subjects

9 patients (26.6 ± 8.3 years, 8 males, 1 female) with unilateral insufficiency of the ACL, which were scheduled for open reconstruction were included in the study. The duration of symptoms or time since injury ranged from six to ten years. Reconstructive surgery was followed by 9 weeks of ambulant physiotherapy, including cast immobilization over the first 7 weeks. During the first 6 weeks of ambulant physiotherapy the patients performed dynamic and isometric strengthening type exercise with their contralateral leg. Thereafter rehabilitation training was carried out until 26 weeks and patients subsequently performed self-motivated physical activity. During the rehabilitation training the strengthening type exercise was supervised and extended to both legs in 3 sessions of 30 min a day. The study protocol has been approved by the ethics committee of the Canton of Bern (Switzerland) and has been performed in accordance with the ethical standards laid down in the 1964 Declaration of Helsinki.

### Design

At time points 0, 52, 260, and 832 weeks respective to reconstructive surgery of the ACL, clinical assessments of knee stability and arthrosis were carried out on the operated leg. At time points 0, 9, 26, and 260 weeks respective to surgery, a computed tomography scan of the quadriceps group was recorded and muscle biopsies were collected under local anesthesia from the belly portion of *vastus lateralis* of the operated and the contralateral leg. Muscle biopsies were split in two parts; one part was processed for the morphometrically analysis of muscle composition based on electron microscopy as described (Luthi et al., 1986). The other part was rapidly frozen in liquid nitrogen-cooled isopentane and stored at -196°C in 1.8 mL cryogenic vials (Nunc, Sigma, Buchs, Switzerland) until being used for the analysis of fiber type distribution or selected molecular regulators of the mitochondrial, myogenic and angiogenic phenotype. Measurements of cellular composition were carried out within months after biopsy collection. Molecular measurements were carried out on the remainders of the frozen samples after 30–35 years of air-tight storage at -196°C. As data from a time point before ACL insufficiency was diagnosed was unavailable, we referenced all measurements to the average values in the contralateral non-operated leg 260 weeks after ACL reconstruction. This sample arguably is more closely related to normal values than those in legs with ACL instability (Gerber et al., 1985; Konishi et al., 2002; Palmieri-Smith et al., 2008; Palmieri-Smith and Lepley, 2015).

### Reconstructive Surgery

Affected knees were subjected to standardized open surgery and grafting. Biopsies were collected intraoperatively from *m. vastus lateralis* with a Bergstroem needle as described (Gerber et al., 1985; Luthi et al., 1986).

### Post-operative Therapy

Patients wore a cast for 7 weeks under stabilization of knee rotation. During that period, the patients were instructed to perform a simple dynamic and isometric strength and flexibility home training of the contralateral non-operated leg. After cast removal, supervised rehabilitation training was performed until week 26. The training was composed of daily isometric and dynamic exercises for strength, stretching and neuromuscular training (starting at week 9) and jogging (starting at week 17). In the following self-motivated physical activity was carried out.

### Clinical Assessment

Routine clinical measurements were performed (Gerber et al., 1985; Domnick et al., 2016; van Yperen et al., 2018). Knee stability and ACL injury was estimated with Lachmann and drawer test, the Pivot shift test, and by a radiological evaluation. Arthrosis was estimated with a standardized index as modified from Ahlbeck and the intraoperative observations based on the outerbridge classification. Knee function was graded according to the Lysholm score and crepitus. A full list of parameters is available in **Supplementary Table S1**.

### Computed Tomography

With the patient supine and the knee flexed to 20 degrees a computed tomographic cross-sectional picture of both limbs was acquired at twenty-five centimeters proximal to the medial joint line using a CT scanner (SOMATOM SF, 125 kV, 230 mAs, 5 s; Siemens, Erlangen, Germany). The cross-sectional area was estimated using a MOP AMO3 semi-automatic electronic planimeter (Kontron AG; Zurich, Switzerland).

### Quantitative Microscopy

Fiber type composition of vastus lateralis muscle was assessed based on the measure of the distribution and cross sectional area of fiber types after histochemical staining for myofibrillar ATPase after alkali or acid incubation as described (Luthi et al., 1986; Berchtold et al., 2000; **Supplementary Figure S1**). The analysis of capillary-to-fiber ratio, and capillary density (as capillaries per area), and muscle ultrastructure (based on the volume density of muscle organelles, i.e., intramyofibrillar, subsarcolemmal and total mitochondria, myofibrils intramyocellular lipids and residual sarcoplasmic volume) was carried out morphometrically based on electron microscopy as described (Luthi et al., 1986). Approximately 400 fibers from each leg were included in the histochemical analysis.

### Protein Biochemistry

Levels of molecular regulators of the myogenic (myoD, myoG) and metabolic phenotype (respiratory complex I-V, tenascin-C, VEGF), and actin, were determined in total homogenate after separation of proteins by SDS/PAGE and immunoblotting and signal detection with enhanced chemoluminescence essentially as described (Frese et al., 2016; Ruoss et al., 2017). The primary antibodies used are provided in **Table 1**. A paired design was applied with the same amount of protein being loaded for all time points after reconstructive surgery in the operated and contralateral leg, respectively, from the same patient in adjacent lanes of the same gel. Recorded signal intensity of the respective band was corrected versus background and standardized to the signal for actin.

**Table 1:** Primary antibodies being employed for immunoblotting.

Antigen	Antibody	Source	Reference
Tenascin-C, monoclonal	B28.13	Prof. Chiquet	Hughes et al., 1999
MyoD	C20 sc-304	Santa Cruz	Li et al., 2013
Myogenin	F5D sc-12732	Santa Cruz	Li et al., 2013
NDUFA9, monoclonal	20C11B11B11	Invitrogen	Andersen et al., 2000
SDHA, monoclonal	2E3GC12FB2AE2	Invitrogen	Andersen et al., 2000
UQCRC1, monoclonal	13G12AF12BB11	Invitrogen	Andersen et al., 2000
COX4I1, monoclonal	20E8C12	Invitrogen	Andersen et al., 2000
ATP5A1, monoclonal	15H4C4	Invitrogen	Andersen et al., 2000
Sarcomeric actin, monoclonal	A2172	Sigma	Andersen et al., 2000
C-terminal FAK		Prof. Ziemiecki	van Yperen et al., 2018
N-terminal FAK	AF4467	R&D systems	
pY397FAK	P00151	Boster Biological Technology	


Levels of Y397 phosphorylation of FAK and of total FAK protein were measured in muscle homogenates using the U-PLEX development system (Meso Scale Discovery). An antibody directed against the N-terminal region of FAK (AF4467, R&D systems) and an antibody that detects the phosphorylation at tyrosine 397 (P00151, Boster Biological Technology) were biotinylated (Biotin-NH_2_ labeling Kit, KT-221, Kamiya Biomedical Company) and used as capture antibodies. The C-terminal FAK antibody was purified from FAK serum “Lulu” [gift of Dr Andrew Ziemiecki, University of Bern (Fluck et al., 1999)] using AbPure Antibody Purification System (Innova Biosciences) and conjugated with SULFO-TAG^TM^ (MSD GOLD SULFO-TAG^TM^ NHS-Ester) to be used as detection antibody. The assay was validated against signals in samples of known FAK activity and amount (Durieux et al., 2009; Klossner et al., 2009, 2013). 20 microgram proteins of each homogenate were analyzed and specific Y397 phosphorylation of FAK was calculated (as signal ratio of FAK Y397 phosphorylation vs. total FAK).

### Statistics

Statistical analysis was carried out with SPSS (IBM). Data were assembled in MS-Excel (Msoffice, Kildare, Ireland). For the evaluation, and display of level alterations, actin-related protein levels, and the values for parameters of cellular composition, were referenced to the values 260 weeks post reconstruction in the contralateral leg, by centering them to the respective mean values. Levels of the mechano-transducer FAK and its Y397 phosphorylation levels were referenced to the mean values 260 weeks post reconstruction in the contralateral leg. Repeated-measures ANOVAs for the repeated factors leg (operated, contralateral) and time (0, 9, 26, 52, 260, or 832 weeks where appropriate) were carried out for the muscle data. Effects were localized with a test for least significant difference and displayed as mean ± standard errors (SE). Linear relationships were assessed based on Kendal Tau correlations, because some comparisons concerned non-parametric data. Significance-weighed r-values (cut off *p* < 0.05) were calculated using MS-Excel (MS Office, Kildare, Ireland) displayed as a correlation matrix using publicly available Cluster and TreeView software essentially as described (Li et al., 2013).

## Results

### Clinical Parameters

Values for the indices of knee stability after reconstructive surgery are shown in **Table 1**. Based on this characterization of laxity and knee stability in the operated leg was improved 52 weeks after reconstructive surgery and remained at a comparable level 260 and 832 weeks after surgery (**Table 2**). The degree of arthrosis remained unchanged between 0 and 52 weeks and tended to worsen between 260 and 832 weeks after surgery.

**Table 2:** Timeline of clinical endpoints.

					p-value	p-values: 0 week vs.		
						
	0 week	52 weeks	260 weeks	832 weeks	Time	52 weeks	260 weeks	832 weeks
VSklin	1.89 ± 0.20	0.78 ± 0.24	1.06 ± 0.21	1.39 ± 0.29	0.024	0.006	0.008	0.172
VSRx	15.67 ± 2.03	7.22 ± 1.08	9.69 ± 1.96	10.56 ± 1.54	0.006	0.021	0.016	0.005
Lachant	1.83 ± 0.29	0.78 ± 0.31	1.06 ± 0.21	1.44 ± 0.26	0.073	0.054	0.030	0.415
Lachpost	0.00 ± 0.00	0.06 ± 0.06	0.28 ± 0.09	0.22 ± 0.15	0.056	0.347	0.013	0.169
Krepit	0.22 ± 0.15	0.44 ± 0.18	0.78 ± 0.15	0.67 ± 0.17	0.180	0.511	0.064	0.047
Axfpat	0.33 ± 0.17	0.11 ± 0.11	0.56 ± 0.18	0.67 ± 0.17	0.270	0.347	0.347	0.081
Swell	0.22 ± 0.15	0.22 ± 0.15	0.22 ± 0.15	0.33 ± 0.11	0.933	1.000	1.000	0.681


*Mean ± SE of assessed clinical endpoints in the operated leg. The endpoints refer to the functional integrity and injury of the ACL and knee joint as revealed through assessing the translation of anterior or posterior bony element after manual manipulation of the knee joint; relative to the contralateral side (Gerber et al., 1985; Domnick et al., 2016; van Yperen et al., 2018. Abbreviations: VSRx, anterior drawer test assessed radiologically; VSklin, anterior drawer test assessed clinically; Lachant, anterior function test (Lachman); Lachpost, posterior function test (Lachman); Krepit, crepitation; Axfpat; degree of arthrosis of the femoro-patellar joint (Ahlbaeck); swell, swelling. Effects were assessed with a repeated-measures ANOVA with least significant differences as *post hoc* test *n* = 9.*

### Plasticity of Extensor Muscle From the Operated Leg

At week 0, the cross-sectional area (CSA) of the quadriceps group in the subsequently operated ACL insufficient leg was 11% lower than the contralateral leg at the same time point and 18% than week 260 (**Supplementary Figure S2**). The quadriceps CSA was further reduced during the 9 weeks of immobilization and ambulant physiotherapy and recovered to reference values in the contralateral leg after week 260.

**FIGURE 1:**
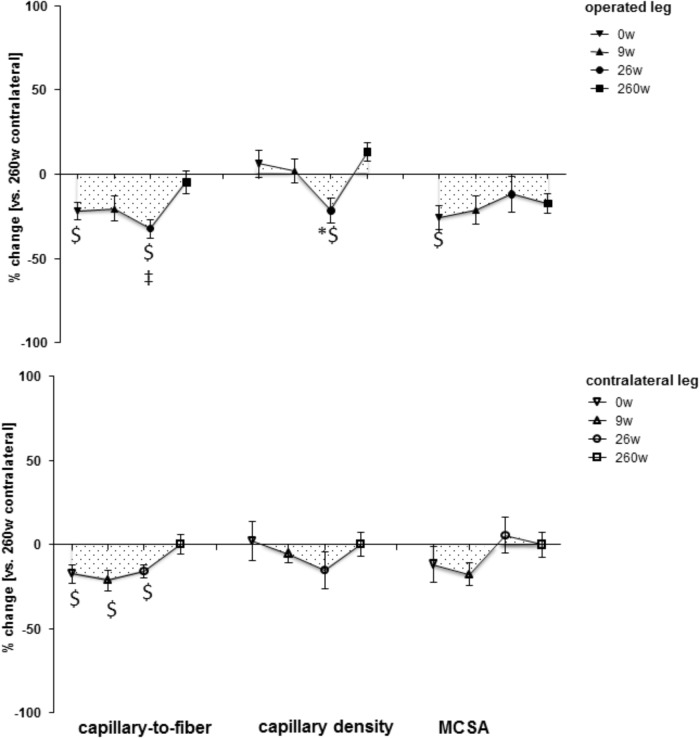
Time line of compositional alterations in *m. vastus lateralis.* Line graph of mean ± SE of the differences in assessed volumetric parameters of muscle composition over time. Values are expressed respective to reference values at 260 weeks in the contralateral leg. The 260-week time point in the contralateral leg served as reference. For details see the list of abbreviations. ^$^, *p* < 0.05 vs. 260 weeks contralateral leg; ^∗^, *p* < 0.05 vs. 0 week same leg; ^‡^, *p* < 0.05 vs. same time point contralateral leg. Repeated-measures ANOVA with *post hoc* test of Fisher (*n* = 9).

**FIGURE 2:**
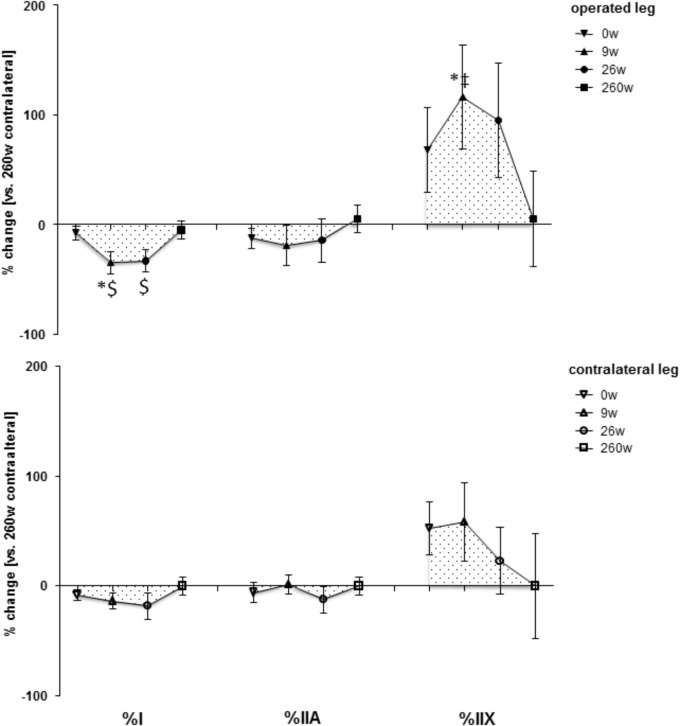
Time line of adjustments in fiber type distribution. Line graph of mean ± SE of the differences in assessed volumetric parameters of muscle composition over time. Values are expressed respective to reference values at 260 weeks in the contralateral leg. For details see the list of abbreviations. ^$^, *p* < 0.05 vs. 260 weeks contralateral leg; ^∗^, *p* < 0.05 vs. 0 week same leg; ^‡^, *p* < 0.05 vs. same time point contralateral leg. Repeated-measures ANOVA with *post hoc* test of Fisher (*n* = 9).

**FIGURE 3:**
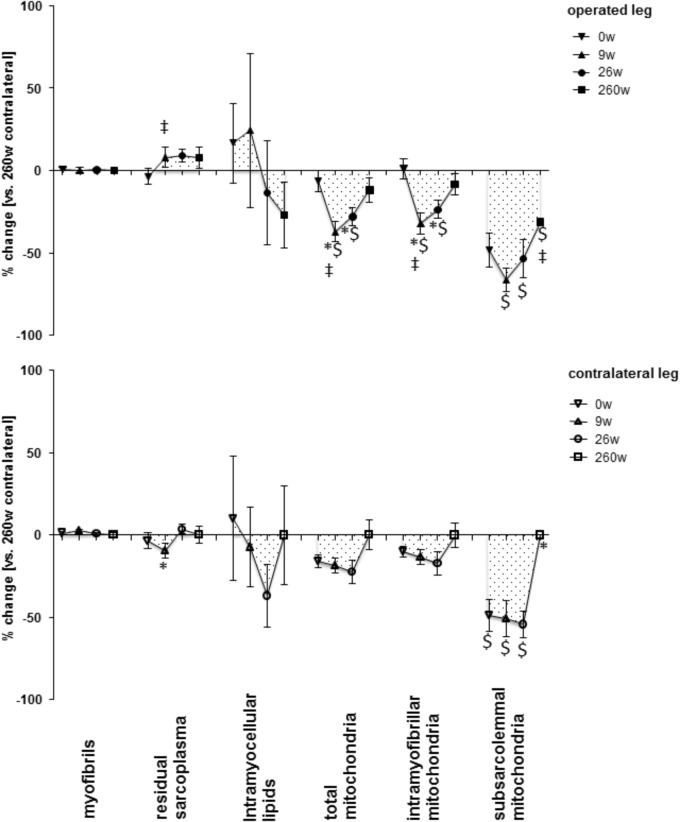
Time line of alterations in muscle fiber composition in *m. vastus lateralis.* Line graph of mean ± SE of the differences in assessed volumetric densities of muscle ultra-structures. Values are expressed respective to reference values at 260 weeks in the contralateral leg. For details see the list of abbreviations. ^$^, *p* < 0.05 vs. 260 weeks contralateral leg; ^∗^, *p* < 0.05 vs. 0 week same leg; ^‡^, *p* < 0.05 vs. same time point contralateral leg. Repeated-measures ANOVA with *post hoc* test of Fisher (*n* = 9).

**Supplementary Figure S1** shows examples of the measured cellular and subcellular (i.e., ultrastructural) parameters in *m. vastus lateralis*. At week 0, the mean cross-sectional area (MCSA) of muscle fibers (-24%) and capillary-to-fiber ratio (-24%) in *m. vastus lateralis*, were lower than reference values in the contralateral leg at week 260 (**Figures 1, 2**). At week 9, the percentage of fast type IIX muscle fibers was increased from 20.6 to 29.5% at the expense of slow type I muscle fibers (49.9 to 40.3%; **Figure 2** and **Supplementary Table S2**). Equally, the volume densities of intramyofibrillar and subsarcolemmal mitochondria were decreased between week 0 and 9, when residual sarcoplasmic volume density was increased (**Figure 3**).

At the 26-week time point after rehabilitation training none of the muscle parameters in the operated leg had recovered. At week 260 most myocellular parameters (capillary-to-fiber ratio, fiber type percentages and the volume density of intramyofibrillar mitochondria, had recovered to the reference values in the contralateral leg at week 260 (**Figures 1–3**). At this endpoint in the operated leg, the volume density of subsarcolemmal mitochondria in *m. vastus lateralis* (*p* = 0.01), was lower than in the contralateral leg; and the MCSA of muscle fibers (*p* = 0.06) demonstrated a trend for lower values than the contralateral reference.

### Expression of Mitochondrial and Myogenic Proteins in Extensor Muscle From the Operated Leg

**FIGURE 4:**
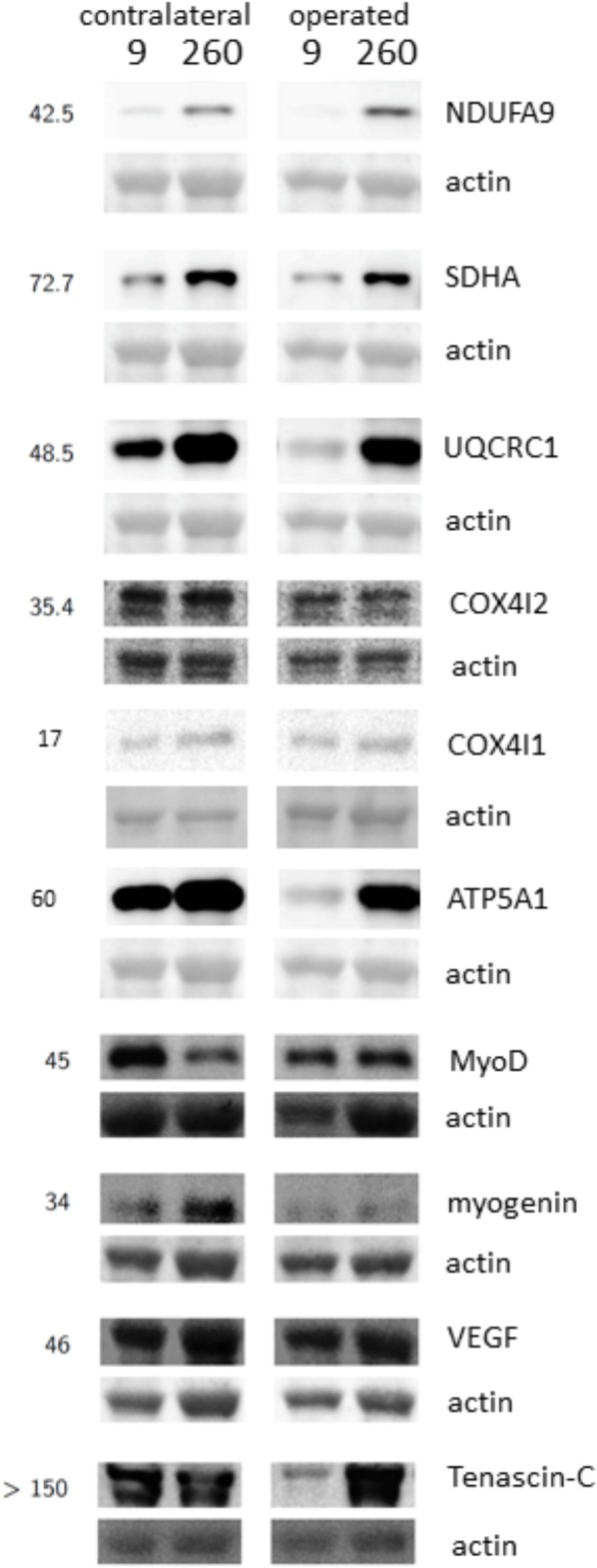
Detection of protein expression. Example immunoblots demonstrating the detection of markers of respiratory complexes I, II, III, IV and V, MyoD, Myogenin, VEGF, and Tenascin-C in *m. vastus lateralis* at the 9-week and 260-week time point after ACL reconstruction in the operated and the contralateral leg. Numbers to the left indicate the appropriate position of molecular weight markers (in kiloDalton).

**FIGURE 5:**
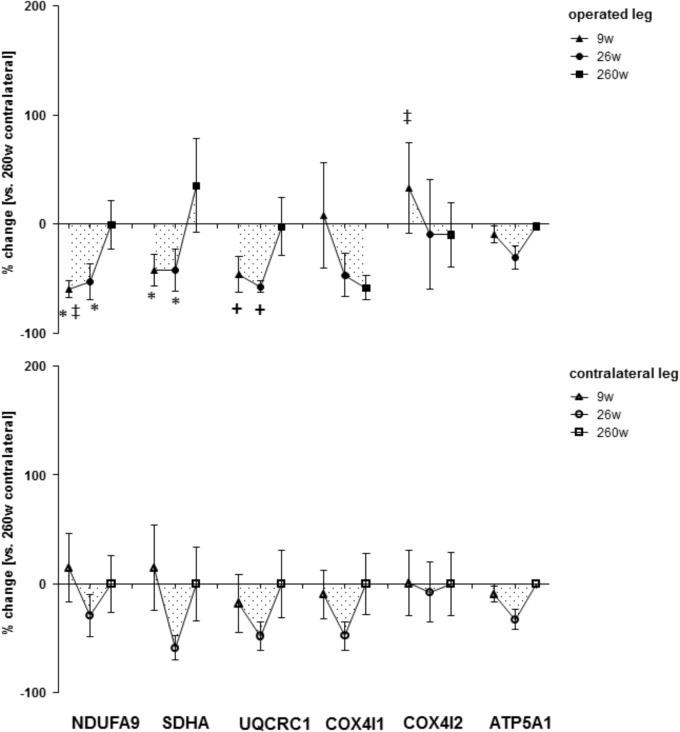
Time line of alterations of respiratory chain markers in *m. vastus lateralis*. Line graph of mean ± SE for the differences of respiratory chain marker proteins (NDUFA9, SDHA, UQCRC1, COX4I1, COX4I2, and ATP5A1) in *m. vastus lateralis*. Values are expressed respective to reference values at 260 weeks in the contralateral leg ^∗^, *p* < 0.05 vs. 260w same leg; ^+^, 0.05 ≥ *p* < 0.10 vs. 260 weeks same leg; ^‡^, *p* < 0.05 vs. same time point contralateral leg. Repeated-measures ANOVA with *post hoc* test of Fisher (*n* = 9).

**FIGURE 6:**
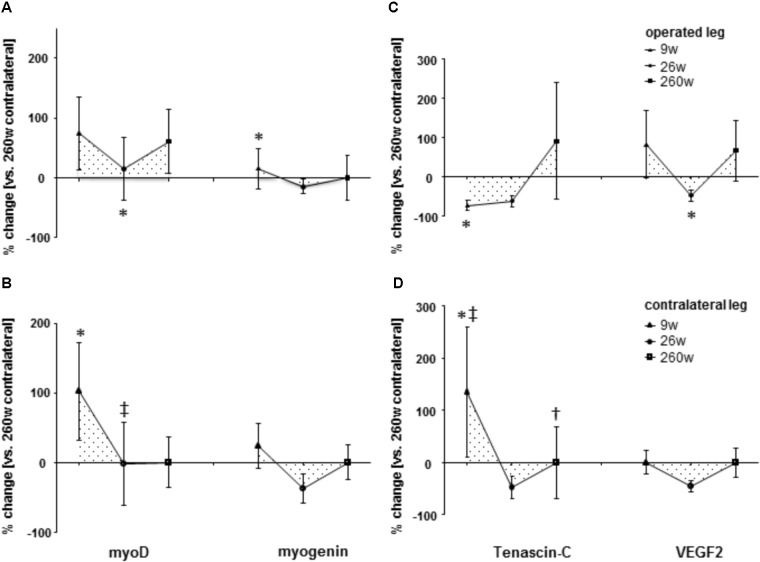
Time line of alterations of myogenic and angiogenic regulation. Line graph of mean ± SE of the differences of molecular regulators of myogenesis [**(A,B)**; myoD and myogenin] and angiogenesis [**(C,D)**; tenascin-C and VEGF2] in *m. vastus lateralis*. Values are expressed respective to reference values at 260 weeks in the contralateral leg. ^∗^, *p* < 0.05 vs. 260 weeks same leg; ^+^, 0.05 ≤ *p* < 0.10 vs. 260 weeks same leg; ^‡^, *p* < 0.05 vs. same time point contralateral leg. Repeated-measures ANOVA with *post hoc* test of Fisher (*n* = 9).

Protein levels of molecular markers of mitochondrial respiration complexes I-III in *m. vastus lateralis* of the operated leg were reduced 9 and 26 weeks after reconstructive surgery and recovered to the reference levels in the contralateral leg at week 260 (**Figures 4, 5**). The level of the myogenic regulator myogenin was increased 9 weeks after surgery compared to 260 weeks, when the tenascin-C protein level was lowered (**Figure 6**).

### Alterations in Extensor Muscle in the Contralateral Leg

The composition of *m. vastus lateralis* in the contralateral leg demonstrated qualitatively similar changes as the operated leg 9 and 26 weeks after reconstructive surgery (**Figures 1–3**). Capillary-to-fiber ratio and subsarcolemmal mitochondria content was reduced at the time point of surgery (week 0) and remained lower until the end of rehabilitation training at week 26 (**Figures 1, 3**). Residual sarcoplasmic volume was reduced in the contralateral leg at 9 weeks after surgery (**Figure 3**).

Protein levels of molecular markers of mitochondrial respiration complexes I-V were not affected by surgery in the contralateral leg (**Figures 4, 5**). At week 9, the marker of complex IV, COX4I2, was more expressed in the operated leg compared to the contralateral leg. At the same time point, the levels of the myogenic regulator myoD and tenascin-c were increased compared to the levels 260 weeks after surgery (**Figure 6**).

### Regulation of the Mechano-Transducer FAK During Rehabilitation

**FIGURE 7:**
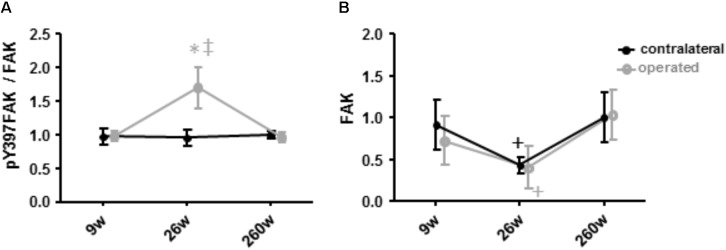
Alterations of Y397 specific FAK phosphorylation and FAK levels. Line graph of mean ± SE of the values for the specific phosphorylation at tyrosine 397 (**A**, phospho397FAK/FAK) and the total level of focal adhesion kinase (**B**, FAK) in *m. vastus lateralis*. Values are expressed respective to reference values at 260 weeks in the contralateral leg. ^∗^, *p* < 0.05 vs. 260 weeks same leg; ^+^, 0.05 ≤ *p* < 0.10 vs. 260 weeks same leg; ^‡^, *p* < 0.05 vs. same time point.

An interaction effect of leg (*p* = 0.044) and leg x time (*p* = 0.026) was identified for the specific phosphorylation of FAK at tyrosine 397 (pY397FAK/FAK) in *m. vastus lateralis*. This was explained by a transient and selective elevation of the specific phosphorylation of FAK at tyrosine 397 in the operated leg at week 26 (+113%, *p* = 0.005; **Figure 7**), differing to the values in the contralateral leg at the same time point (*p* = 0.004).

The levels of Y397 phosphorylated FAK (pY397FAK) and FAK in *m. vastus lateralis* tended to decrease by 48% (*p* = 0.091) and 66% (*p* = 0.056) at week 26. At 260 weeks both values were increased again to levels comparable to those seen at week 9 (*p* = 0.379 and *p* = 0.456). Level alterations of pY397FAK and FAK did not differ between the contralateral and the operated leg (*p* = 0.969 and *p* = 0.784, respectively).

### Interrelationships

Linear relationships were identified within and between molecular and cellular parameters, and between molecular and cellular parameters, characterizing muscle composition over both legs and every time points (**Supplementary Figure S3**). Correlations were identified between mitochondria, fiber size and fiber type percentage and capillary related muscle parameters with a set of clinical parameters being related to knee stability (Menmed, Pivot, TMEpreop, patella; **Supplementary Figure S4**).

**FIGURE 8:**
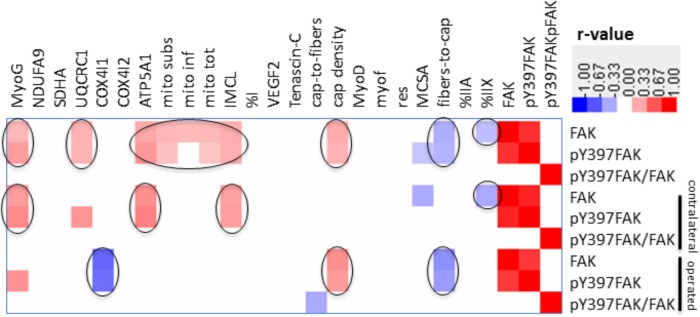
Correlation matrix of relationships between FAK and muscle parameters. Heat map showing *p*-value weighed correlations between molecular and cellular parameters of *m. vastus lateralis* and FAK-related parameters for legs combined or separated. The color code used to visualize *r*-values is given. Only those relationships were shown which met a *p*-value ≤ 0.05. Relationships of interest are circled.

There were also correlations between muscle parameters and the total level of FAK, pY397FAK, and pY397FAK/FAK (**Figure 8**). Over the muscles of both legs, FAK and pY397FAK levels correlated positively with the level of MyoG, mitochondria content and the level of the marker of mitochondrial respiration complexes III and IV, intramyocellular lipids and capillary density. Negative correlations were found with the fiber-to-capillary ratio and the percentage of type IIX muscle fibers. The correlations between FAK and pY397FAK, mitochondrial parameters and type IIX muscle fibers were preserved in the contralateral leg but lost in the operated leg. In the operated leg, capillary density correlated positively, and the level of the marker for mitochondrial complex IV and the fiber-to-capillary ratio correlated negatively to FAK and pY397FAK levels. pY397FAK/FAK levels were negatively correlated with the capillary-to-fiber ratio.

## Discussion

The present investigation focused on the timeline of plasticity in the knee extensor, *m. vastus lateralis*, during recovery from ACL insufficiency after open reconstruction of the ruptured ACL. Based on quadriceps CSA and muscle MCSA in *vastus lateralis*, atrophy was present in the leg suffering for years from ACL-insufficiency and was aggravated concomitantly with a reduction in slow oxidative characteristics in *m. vastus lateralis* during the 9 weeks after surgery under cast immobilization and physiotherapy. Atrophy and compositional adjustments in the *m. vasti* were qualitatively similar between the operated and contralateral legs of patients during the first 9 weeks of ambulant physiotherapy and successive rehabilitation up to week 26. However, the deterioration of the volume density of subsarcolemmal mitochondria, capillary-to-fiber ratio and fiber type distribution was more pronounced in the operated leg. At week 260 after voluntary physical activity, but not at week 26 after rehabilitation training, the structural hallmarks of the mechanical and metabolic phenotype of *m. vastus lateralis* in the operated leg were reestablished respective to reference values in the contralateral leg. The exception was the volume density of subsarcolemmal mitochondria and MCSA of muscle fibers. The findings highlight that the reestablishment of knee extensor muscle composition (and function) in chronically ACL-insufficient patients is a slow process (Ardern et al., 2011), which may not be complete after standard rehabilitation training. The similarity of adjustments was contrasted by different expressional alterations of mitochondrial protein and myogenic regulators in *m. vastus lateralis* between the operated and contralateral leg, at week 9 for NDUFA9, COX4I2, and tenascin-C, and at week 26 for myoD and the specific Y397 phosphorylation of FAK. These molecular observations point to a different muscle response in the early rehabilitation between the operated and contralateral leg when they did undergo a different treatment.

The adjustments of *m. vastus lateralis* composition for the operated leg at time points 0 and 9 weeks, respective to the 260-week time point after reconstructive surgery in the contralateral leg, emphasized the deconditioning of muscle structure (**Figures 1, 2**). The observed lower values for the muscle fiber MCSA and quadriceps CSA in the ACL insufficient leg before reconstructive surgery relates to the reported loss in muscle force as reported for ACL insufficiency (Gerber et al., 1985). Meanwhile, the loss of mitochondria and capillarization in the 9 weeks after reconstructive surgery, including 7 weeks of cast immobilization, is indicative of a reduced fatigue resistance (**Figures 1, 2**). Concomitantly the percentage of fast type IIX muscle fibers, with a typically lowest content of mitochondria and lowest fatigue resistance (David et al., 2004), was increased. Collectively the observed (ultra-) structural adaptations of extensor muscle recapitulate the deconditioning of the slow aerobic muscle phenotype with disuse (Fluck and Hoppeler, 2003). Interestingly, total mitochondrial volume density appeared to be much more sensitive to immobilization than the distribution of muscle fiber types, capillarization and the mean cross sectional area of muscle fibers. A possible role of disuse for the observed muscular reactions is corroborated by the reversion of the structural characteristics in extensor muscle 251 weeks after resumption of weight-bearing muscle activity in the operated leg (**Figures 1, 2**).

The contribution of disuse to muscle deconditioning with ACL insufficiency is supported by a reduction in capillary density and volume density subsarcolemmal mitochondria in *m. vastus lateralis* of the contralateral leg at the time point 0, which continued to be reduced 9 and 26 weeks after surgery (**Figure 1**). Both parameters reflect inter-related characteristics that sustain the elevated metabolic flux in skeletal muscle during exercise (Desplanches et al., 2014). The notion of their interdependence is supported by the identified correlation between both parameters (*r* = 0.60; **Table 1**). Muscle reactions in the contralateral leg, involving alterations in the distribution and MCSA of muscle fiber types, have been shown to occur in the rat in response to the severe surgical interventions of nerve or muscle transplantation (Klossner et al., 2013). By contrast, in our investigation the contralateral leg did not demonstrate a significant alteration in the distribution or MCSA of fiber types in *m. vastus lateralis* (**Figures 1, 2**). These observations emphasize that the *vastus lateralis* muscle of the contralateral leg primarily undergoes metabolic deconditioning with ACL insufficiency.

Our observations point out the time course and magnitude of extensor muscle reconditioning during standard rehabilitation training. Intriguingly 17 weeks of rehabilitation training was ineffective to provoke cellular alterations in muscle composition related to metabolic characteristics and the percentage of slow type fibers was further reduced. In this regard, it is insightful to compare the adaptations in ultra-structure in the studied knee extensor after further 234 weeks of self-motivated activity to the alterations with a few weeks of resistance or endurance type training. For instance, in healthy subjects it has been documented that 6 weeks of an intense bicycle training stimulus produces an increase in mitochondrial volume density (Fluck and Hoppeler, 2003), which in relative terms equals the changes observed for the patients in the present study, 260 weeks after rehabilitative training (**Figure 3**). Similarly, the reported percentage increase in fiber MCSA after 6 weeks of resistance training is equivalent to the changes identified 260 weeks after reconstructive surgery (**Figures 1, 2**) (Fluck and Hoppeler, 2003). Consequently, the reestablishment of normal muscle characteristics in the studied patients after six to ten years of ACL insufficiency appeared to take considerably longer than offered with the selected rehabilitation program. In this regard, quadriceps CSA and muscle fiber MCSA in the *m. vastus lateralis* from the operated leg still tended to be lower than the reference values in the contralateral leg at week 260 (**Supplementary Figure S2** and **Figure 1**). The cellular evidence for impaired mechanical and metabolic function in knee extensor muscle of both legs is of interest in relation to the reported long term consequences of ACL insufficiency and reconstruction that may lead to knee joint degeneration (Lohmander et al., 2007; Noehren et al., 2013).

Increases in the volume density of muscle mitochondria and myofibrils are quantitatively related to the impacting metabolic and mechanical stimulus applied onto the muscle (Fluck and Hoppeler, 2003). The question therefore arises whether training load and volume during rehabilitation was too low to produce the expected adaptations, or whether an intrinsic incapacity of the operated patients with the symptomatic ACL insufficiency did not allow that the expected muscle plasticity manifested (Nyland et al., 2010).

The similarity in adaptations of the *m. vasti* from the operated and contralateral leg is astonishing as one may expect that the contralateral leg will experience a higher load than the functionality impaired operated leg to warrant control of posture when standing or walking. Evidence for such a difference was in fact identified based on the increased levels of the pro-myogenic factor tenascin-C in the contralateral leg after ambulant physiotherapy at week 9, which comprised strengthening type exercise for this leg (**Figure 6**). Tenascin-c expression in skeletal muscle is upregulated by mechanical loading [reviewed in (Crameri et al., 2007; Valdivieso et al., 2017)]. The level increases of tenascin-c protein in *m. vastus lateralis* from the contralateral leg therefore provide molecular evidence for the initiation of a regenerative response in extensor muscle, due to possibly compensatory neuromuscular reactions (Lu et al., 1999; Crameri et al., 2007). As well, we identified elevated levels of the mitochondrial protein COX4I2 within *m. vastus lateralis* of the operated leg at week 9 (**Figure 5**). This protein is known for its hypoxia-sensitive expression in skeletal muscle after single and repeated exercise in inverse relation to contraction-induced capillary perfusion (Desplanches et al., 2014; van Ginkel et al., 2016). Its higher levels in *m. vastus* from the operated leg being suggestive of an important under-perfusion of extensor muscle during the episodes of muscle activation with ambulant physiotherapy.

We also detected selectively elevated specific Y397 phosphorylation of FAK in the operated leg immediately after the 17 weeks of rehabilitation at week 26 when it was not affected in the contralateral leg (**Figure 7**). In rat muscle, 397 phosphorylation levels of FAK are transiently increased after the impact of mechanical loading on skeletal muscle in proportion to time under tension in rat muscle (Klossner et al., 2013; Rahnert and Burkholder, 2013). We interpret the selective increase in Y397 phosphorylation levels as to reflect differences in the onset of resistance type training between the operated and contralateral leg. Intriguingly, at this time point FAK protein levels tended to be similarly decreased in *m. vastus* from both the operated and contralateral leg. This finding is consistent with the down-regulation of FAK expression in human anti-gravity muscle after weeks-to-months of bedrest and space-flight and the lowered FAK protein levels in fast type IIX muscle fibers with a lower degree of recruitment (Fluck et al., 2002; Li et al., 2013; Rittweger et al., 2018). Our present novel finding in humans confirm the downregulation of FAK levels as a possibly critical event associated with muscle unloading (**Figure 7**).

Reductions of mitochondrial protein levels reflect an altered balance between the degradation and synthesis of mitochondrial protein (Booth, 1991; Booth et al., 1996). In this respect a reduction in the synthesis rates of mitochondrial proteins is possibly critical because this depends on contractile activity (Booth, 1991; Booth et al., 1996). The experimental enhancement of FAK protein and Y397 phosphorylation levels in rodent skeletal muscle by somatic overexpression and augmented loading has been shown to increase the expression of mitochondrial transcripts and proteins and type I myosin heavy chain (Durieux et al., 2009), and promotes ribosomal biogenesis (Durieux et al., 2009; Klossner et al., 2009). The interrelationship between FAK and aerobic and contractile characteristics of the slow oxidative muscle phenotype is supported by the in here observed correlation between the levels of FAK and Y397 phosphorylated FAK with the volume densities of mitochondria, expression levels of the markers for mitochondrial respiration complexes III and IV, the myogenic transcription factor myoG which governs oxidative metabolism in mouse muscle [reviewed in (Hughes et al., 1999; Fluck and Hoppeler, 2003)], and the percentage of the IIX muscle fibers which are typically of a fast-glycolytic type (Fluck and Hoppeler, 2003; **Figure 8**). As well we identified an increased specific Y397 phosphorylation of FAK in *m. vastus lateralis* from the pathologic leg after the resistance type rehabilitation stimulus at week 26, when the down-regulation of mitochondrial protein levels was halted (**Figure 4**). In conjunction with the not improved muscle capillarization before week 260, it appears, however, that *m. vastus lateralis* of the operated leg did not receive sufficient recruitment-related stimuli during the 17 weeks of rehabilitation training to allow the reestablishment of aerobic parameters (Andersen et al., 2000; Fluck and Hoppeler, 2003).

The contention of an insufficient impact of physiological cues to preserve the metabolic muscle phenotype is supported by the minimal effects of rehabilitation on muscle capillarization. Recovery of normal values for the volume density of subsarcolemmal pool of mitochondria, which is a marker of mitochondrial biogenesis [reviewed in (Desplanches et al., 2014)] was even incomplete at week 260. Potentially this is reflected by the reduced protein level of markers of mitochondrial respiratory chain IV, COX4I1, which is associated with the subsarcolemmal pool of mitochondria (Desplanches et al., 2014), and which tended to not have reached the reference levels at week 260 (*p* = 0.12; **Figure 5**). In this regard, complementary endurance type stimuli would be worth considering as they produce considerable gains in subsarcolemmal mitochondria in *m. vastus lateralis*, over a course of six weeks in healthy subjects (Desplanches et al., 2014).

The further observed linear relationship between a connected entity of clinical indices of knee stability (Menmed, pivot, TMEpreop, patella) at the time point of reconstruction and cellular hallmarks of muscle composition highlights that biological relationships within soft- and hard tissue of the knee joint do exist. This suggests that hard tissue factors may set the degree of functional recovery of muscle characteristics (such as force production and metabolic supply) that can be achieved in the first phase of recovery with physical activity.

An important aspect of our study was that we identified that the slow-to-fast fiber transition of muscle fiber types in knee extensor muscle was reversible (**Figure 2**). Apart from investigations with disused muscle in laboratory species (Desplanches et al., 1987; Pette et al., 2002), the former phenomenon has to the best of our knowledge only been described in one human study with 12-weeks of strength training of young subjects after 3 weeks of immobilization (Howald, 1982; Hortobagyi et al., 2000; Fluck and Hoppeler, 2003; Wilson et al., 2012). Our findings now indicate that the reestablishment of the normal fiber type distribution in ACL-insufficient patients in the physiological context of standard rehabilitation and self-motivated exercise may take years to complete.

Our study has several limitations: *Vastus lateralis* muscle is not the main muscle operating extension of the knee and stabilizing the knee joint during twist and turns (Narici et al., 1992). Thermal measurements, however, indicate that this muscle serves as a good proxy for the activity of the quadriceps group during knee extension (Krustrup et al., 2004), and it has been noted that *m. vastus lateralis* did undergo the most pronounced atrophy of all quadriceps muscles with insufficiency of the ACL (Gerber et al., 1985). The measured changes in the quadriceps CSA support the microscopy-based conclusions on the reversibility of reductions in fiber MCSA for the *m. vastus lateralis* with disuse and rehabilitation, although the anatomical alterations in the single muscle may not be fully represented by the CT-based measures for CSA of the entire knee extensor group. To encounter for the level of physical activity we quantified the protein level and Y397 phosphorylation of the phosphotransferase FAK, which serve as a proxy for chronic and acute alterations in muscle loading (Fluck et al., 2002; Li et al., 2013). While the identified transient alterations in Y397 phosphorylation and protein on FAK are in support of an insufficient degree of impact during rehabilitation, additional metabolo- and mechano-regulated signaling processes (Hoppeler et al., 2011; Egan and Zierath, 2013), would remain to be investigated to expose the molecular mechanism underlying muscle reconditioning in the clinical setting of rehabilitation. Due to the minimally invasive sampling of muscle biopsies, an insufficient amount of tissue, however, remained after the cellular characterization, to allow measures of protein levels for the sample point being collected during reconstructive knee surgery. Also, clinical parameters were not recorded for the contralateral leg, as this is not a custom measurement in an orthopedic clinic. Thus, the latter type of data is underpowered to disentangle whether clinically assessed alterations of the knee joint during rehabilitation from ACL reconstruction are due to surgery and physical activity or the individual musculoskeletal biology of the patient. Last, but not least, the open reconstructive surgery, and the post-operative cast immobilization (Smith and Davies, 2008), deployed does not correspond to the state-of-art to address the studied clinical problem, which in instances is treated nowadays in a conservative manner despite a similar long term outcome as a surgical approach (Culvenor et al., 2013; van Yperen et al., 2018). Within these mentioned boundaries, our manuscript provides novel relevant insight on the molecular and cellular mechanism underling the course of recovery of knee extensor function after open knee surgery.

## Conclusion

While the results of our longitudinal investigation must be validated in a larger cohort, we provide the first evidence that cellular reactions in knee extensor muscle after chronic ACL insufficiency and open reconstruction are qualitatively similar in the operated and contralateral leg and related to disuse. Standard rehabilitation training was incomplete to produce recovery of the normal fiber type distribution, muscle fiber MCSA, capillarization, mitochondrial volume density and markers of mitochondria respiration, but selectively elevated the activation status of the proxy of mechano-transduction, FAK, in the operated leg, when its protein level was reduced. Linear relationships between the levels of the activated mechano-transducer FAK and mitochondrial parameters, hint that adjustments in the metabolic phenotype of the knee extensor *m. vastus lateralis* are graded to the impact of mechanical cues during the phases of ambulant physiotherapy, rehabilitation training and self-motivated physical activity. Leg differences in myogenic and hypoxia-related protein expression further support the role of mechanical and metabolic factors as explanation for the observed early differences in plasticity of extensor muscle during rehabilitation. A lower volume density of subsarcolemmal mitochondria in the operated leg respective to the contralateral leg, emphasizes that self-motivated physical activity over 5 years after reconstructive surgery, was not efficient to promote full reestablishment of metabolic functioning in the operated, formerly ACL-insufficient, leg.

## Author Contributions

MF, MB, J-ML, HH, and CG conceived and designed the study. CV, AB, SK, SR, PV, HC, and HH performed the experiments. MF, CV, AB, PV, J-ML, MB, and CG analyzed the data. MF, MVF, HH, and CG interpreted the results. CG acquired funding. MF, CV, AB, and SK prepared the figures. MF, CV, and HH drafted the manuscript. MF, SR, and MVF edited the manuscript.

## Conflict of Interest Statement

The authors declare that the research was conducted in the absence of any commercial or financial relationships that could be construed as a potential conflict of interest.

## Abbreviations

ACL: anterior cruciate ligament
ATP5A1: ATP synthase subunit alpha
COX4I1: cytochrome c oxidase subunit 4 isoform 1
COX4I2: cytochrome c oxidase subunit 4 isoform 2
CSA: cross-sectional area
FAK: focal adhesion kinase
MCSA: mean cross-sectional area
myoD: myoblast determination protein 1
myof: myofibrils
myoG: myogenin
NDUFA9: NADH dehydrogenase ubiquinone 1 alpha subcomplex subunit 9
pY397FAK: Y397 phosphorylated FAK
pY397FAK/FAK: specific phosphorylation of FAK at tyrosine 397
SDHA: succinate dehydrogenase A
UQCRC1: ubiquinol-cytochrome c reductase complex subunit 1
VEGF: vascular endothelial growth factor
Y397: tyrosine 397
