# Supplementary material for: Knee Extensors Muscle Plasticity Over a 5-Years Rehabilitation Process After Open Knee Surgery
Source: Front Physiol. 2018 Sep 25;9:1343. doi: 10.3389/fphys.2018.01343 (PMC6178139; doi:10.3389/fphys.2018.01343)
Supplement: Supplementary file 1 [file Image_1.pdf]

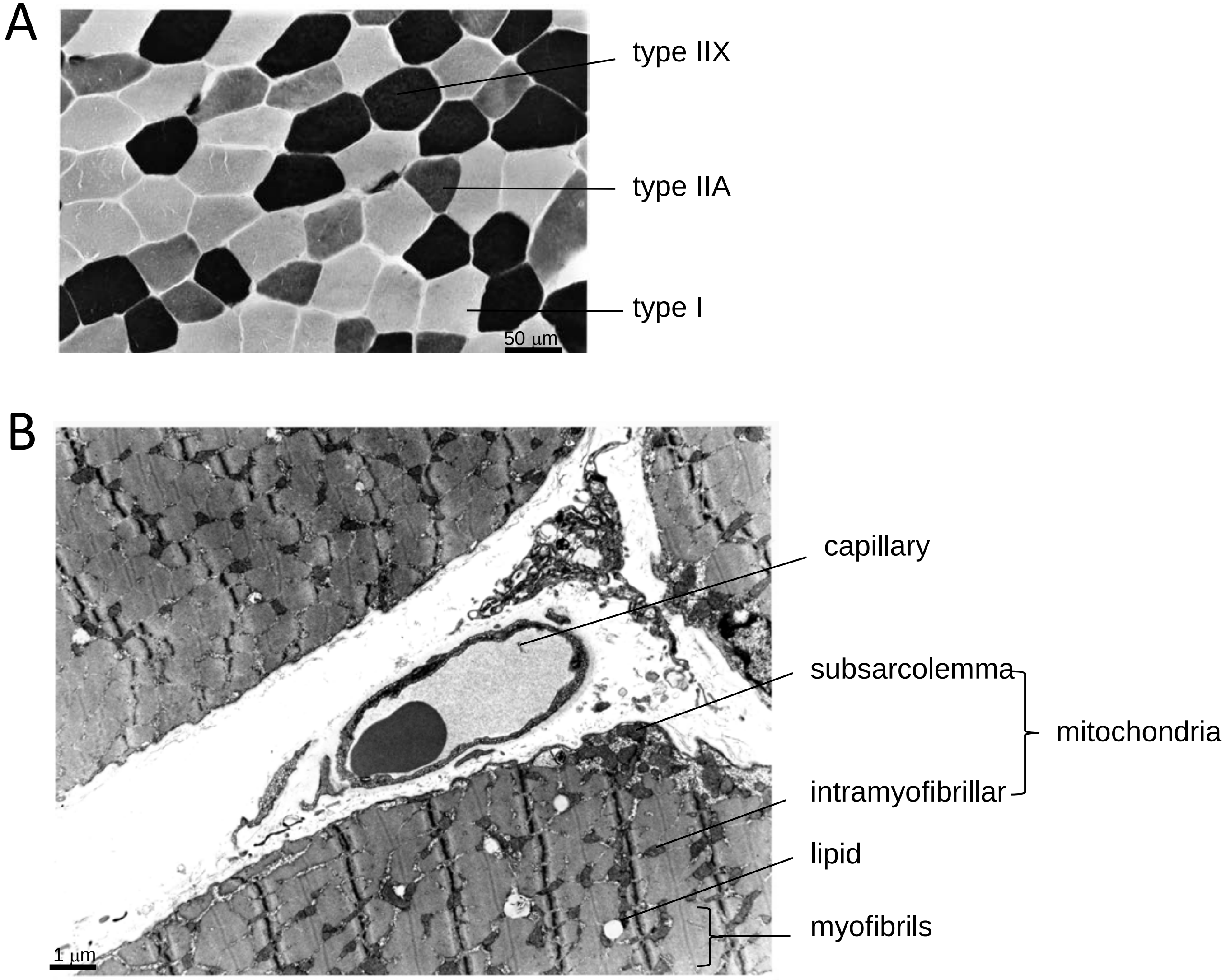

Supplementary Figure S1: *Illustration of measured cellular parameters*. A,B) Photographic record of alkali stable myofibrillar ATPase in cross-sectioned muscle fibers (A) and electron micrograph (B) from a vastus lateralis biopsy of a contralateral control. Examples of the quantified cellular and ultrastructural parameters are indicated.
