# Supplementary material for: Knee Extensors Muscle Plasticity Over a 5-Years Rehabilitation Process After Open Knee Surgery
Source: Front Physiol. 2018 Sep 25;9:1343. doi: 10.3389/fphys.2018.01343 (PMC6178139; doi:10.3389/fphys.2018.01343)
Supplement: Supplementary file 2 [file Image_2.pdf]

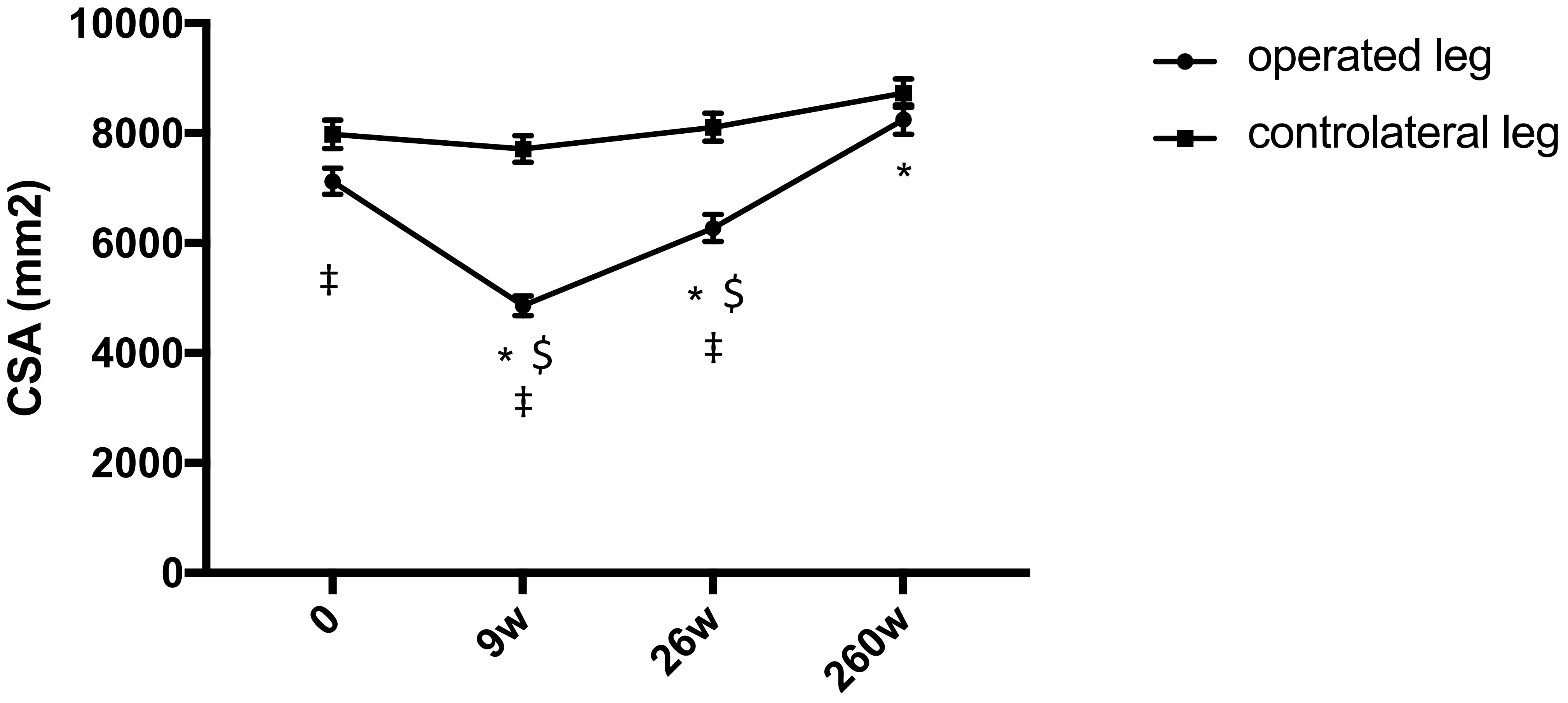

Supplementary Figure S2: *Time line of compositional alterations in the knee extensor group*. Line graph of mean ± SE of the cross-sectional area (CSA) of the quadriceps group in the ACL insufficient and contralateral leg over time. $, p < 0.05 vs. 260w contralateral leg; *, p < 0.05 vs. 0w same leg; ‡, p < 0.05 vs. same time point contralateral leg. Repeated-measures ANOVA with post-hoc test of Fisher. N=9.
