# Supplementary material for: Knee Extensors Muscle Plasticity Over a 5-Years Rehabilitation Process After Open Knee Surgery
Source: Front Physiol. 2018 Sep 25;9:1343. doi: 10.3389/fphys.2018.01343 (PMC6178139; doi:10.3389/fphys.2018.01343)
Supplement: Supplementary file 3 [file Image_3.pdf]

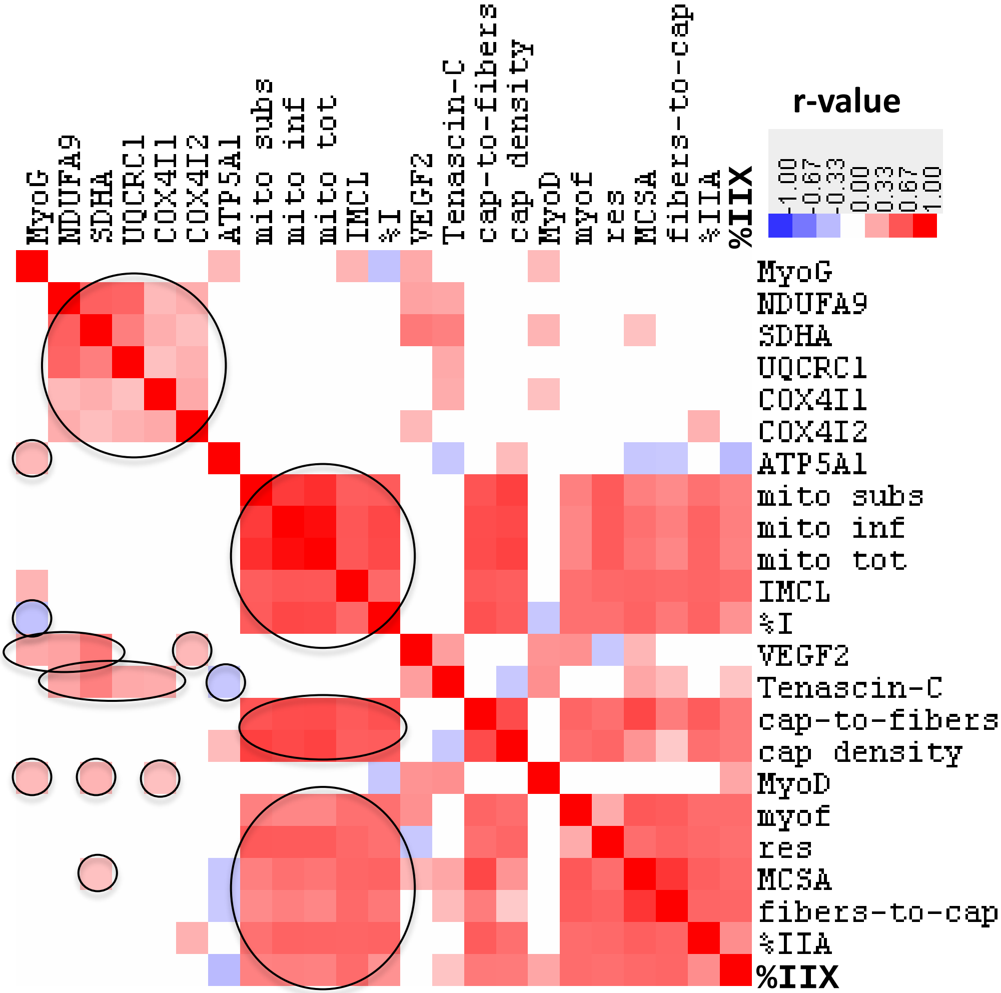


***Supplementary Figure S3:** Correlation matrix of relationships between muscle parameters*. Correlation matrix of relationships between muscle parameters. Heat map showing p-value weighed correlations between molecular and cellular parameters of m. vastus lateralis for legs combined and over every time point. The colour code used to visualize r-values is given. Only those relationships were shown which met a p-value ≤0.05. Relationships of interest are circled.
