# Supplementary material for: Knee Extensors Muscle Plasticity Over a 5-Years Rehabilitation Process After Open Knee Surgery
Source: Front Physiol. 2018 Sep 25;9:1343. doi: 10.3389/fphys.2018.01343 (PMC6178139; doi:10.3389/fphys.2018.01343)
Supplement: Supplementary file 5 [file Table_1.docx]

***Supplementary table S1:*** *Clinically assessed parameters.*

*Parameter abbreviation type of data*

anterior drawer test with knee in 90°flexion VSRx continuous (mm)

anterior drawer, radiologically assessed VSklin ordinal (0-3)

posterior drawer test with knee in 90°flexion HSRx continuous (mm)

posterior drawer, radiologically assessed HSklin ordinal (0-3)

medial stability with knee in 30°flexion aufmed ordinal (0-3)

lateral stability with knee in 30°flexion auflat ordinal (0-3)

anterior function (Lachman) Lachant ordinal (0-3)

posterior function (Lachman) Lachpost ordinal (0-3)

Swelling Swell ordinal (0-1)

Pivot shift test pivot ordinal (0-1)

osteoarthritis femoro-patellar joint (Ahlbaeck), radiological Axfpat ordinal (0-1)

Painful crepitus Krepit ordinal (0-1)

Knee function (Lysholm) Lyshol ordinal (0-100)

Cartilage damage tibial bone (Outerbridge) Tibiamed ordinal (0-4)

Damage femoral bone (Outerbridge) Femurmed ordinal (0-4)

Arthrosis degree Ahlbae ordinal (0-4)

Patella integrity (outerbridge) patella ordinal (0-4)

Giving way Gway ordinal (0-1)

lateraler meniscus integrity Menlat ordinal (1-3)

medial meniscus integrity Menmed ordinal (1-3)

knee pain pain ordinal (0-1)

extension Ext continous 0°-10°

flexion Flex continous (125°-160°)

blokade block ordinal 0-1)

physical activity (sports) sport ordinal (0-3)

subjective knee score SubP ordinal (0-100)

subjective knee score contralateral GsubP ordinal (0-100)

partial meniscetomy TMEpraeOP ordinal (0-1)

re-operation ReOP ordinal (0-2)
