# Supplementary material for: Knee Extensors Muscle Plasticity Over a 5-Years Rehabilitation Process After Open Knee Surgery
Source: Front Physiol. 2018 Sep 25;9:1343. doi: 10.3389/fphys.2018.01343 (PMC6178139; doi:10.3389/fphys.2018.01343)
Supplement: Supplementary file 6 [file Table_2.docx]

***Supplemental table S2:*** *Fiber type distribution at the reference point.* Mean ± of standard error of the percentages of type I, type IIA and type IIX muscle fibers in *m. vastus lateralis* from the contralateral leg at 260 weeks. N=9.

**type I type IIA type IIX**

54.8 ± 4.4 % 33.2 ± 2.8 % 12.1 ± 5.7 %
